# Elastic, optoelectronic and photocatalytic properties of semiconducting CsNbO_3_: first principles insights

**DOI:** 10.1038/s41598-023-36875-x

**Published:** 2023-06-23

**Authors:** M. Monira, M. A. Helal, M. N. H. Liton, M. Kamruzzaman, S. Kojima

**Affiliations:** 1 Department of Physics, Begum Rokeya University, Rangpur, Rangpur, 5400 Bangladesh; 2 Department of Physics, University of Rajshahi, Rajshahi, 6400 Bangladesh; 3 Graduate School of Pure and Applied Sciences, University of Tsukuba, Tsukuba, Ibaraki 305-8573 Japan

**Keywords:** Materials for devices, Theory and computation

## Abstract

The cubic phase of CsNbO_3_ (CNO) perovskite has been hypothesized to investigate the elastic, electronic, photocatalytic, and optical properties for various technological applications using first-principles method. The pressure dependent structural stability has been confirmed from computed elastic constants. Relatively high value of elastic moduli, large hardness and toughness suggested that CNO would be applicable to design industrial machineries. The ductile to brittle transition is noticed at 20 GPa. The indirect bandgap of CNO proclaims its suitability for photovoltaic and IR photodetector applications. The total and partial density of states are calculated to show in evidence the contribution of individual atomic orbitals in the formation of bands. The pressure changes orbitals hybridization which can be substantiated by the change in the bandgap. Strong covalency of the Nb–O bond and antibonding character of Cs–O have been anticipated by the Mulliken population analysis and by the contour maps of electron charge density. The low carrier effective mass and high mobility carriers predict the good electrical conductivity of the material. The calculated values of conduction and valance band edge potential illustrate the excellent water-splitting and environmental pollutants degradation properties of CNO.

## Introduction

ABO_3_-type perovskites, a large family of ternary oxides, continue to draw significant attention as materials for solid oxides fuel cells^1^, catalytic^2^ and electrochemical applications^3^, hydrogen membranes^4^, actuators, sensors^5^, etc. Such types of materials are found of great interest because of their adaptable material characteristics and tunable physical properties. These compounds exhibit structural distortions as well as various spin orderings^6^ depending on temperature and pressure. Furthermore, the perovskite’s structural phase transitions^7^, which are crucial for understanding the quantum fluctuations that are driven by the characteristics of the materials may result in fascinating ferroelectrics phenomenon. The perovskite materials also display extraordinary physical properties in electronic devices, like diodes, transistors, integrated circuits^1^, thin-film transistors for flat-panel displays^8^, spin-dependent transport^9^, strong electron lattice couplings^10^, etc.

Recently, new catalysts and photocatalyst materials have gained a lot of attention in wastewater treatment in an effort to reduce some significant challenges connected to heavy ions, dyes, and emerging contaminants. Researchers have been working consistently over the past few decades to create photocatalysts that are stable, effective, affordable, and non-toxic for use in water splitting and environmental remediation. The exceptional stability of ABO_3_-type oxide photocatalysts, which is the most fundamental and crucial prerequisite of photocatalysts for practical application, makes them highly valuable for study^2^. However, the wide bandgap, which were typically brought on by the low valence bands and were composed of O-2*p* orbitals with low potential vs *NHE* (Normal Hydrogen Electrode), frequently served as an intrinsic restriction on the ability of oxide photocatalysts to utilize visible light^11^. Additionally, the generated visible light driven oxide photocatalytic activity remained modest^12^. The improvement of visible light driven photocatalytic activity and the expansion of light response ranges should therefore be the main research areas for oxide photocatalysts^13^. A number of theoretical and experimental research have been done to look at the different characteristics of perovskite systems such as niobates, titanates, cuprates, etc. Very recently, pressure-dependent theoretical investigations on mechanical, electronic, and optical properties of BaCuO_3_^14^, CaCuO_3_ and SrCuO_3_^15^ have been explored which shows their utility for several optoelectronic applications. The improvement of visible light driven photocatalytic activity and the expansion of light response ranges should therefore be the main research areas for oxide photocatalysts^13^. For the construction of lasers and detectors, many semiconducting materials are used^16^. Recent researches have shown the structural, magnetic, and magnetocaloric properties of niobates under pressure^17^. Materials must be customized for individual application since their qualities determine how effective they are in field service.

Nowadays, many theoretical investigations have been performed on niobate oxides such as LiNbO_3_, NaNbO_3_, KNbO_3_, SrNbO_3_, and RbNbO_3_^18^, RbSr_2_Nb_3_O_10_^19^ etc. as well as the temperature-dependent investigations of different properties of CsNbO_3_ have also been demonstrated^17^. These explorations are proved to be essential to the material engineering sectors. These oxides exhibit structural phase transitions at various pressures^20^ and have a high dielectric constant^21^, high breakdown strength^22^, wide bandgap^23^, and low current leakage current density^24^. As a function of temperature, pressure, and particle size, NaNbO_3_ displays a very intricate sequence of structural phase changes^25^. Under light illumination, KNbO_3_ is very stable and nontoxic^26^. Due to its accessibility, low toxicity, and ability to exhibit strong polarization at high electric fields, AgNbO_3_^27^ is another alluring lead-free ferroelectric material. Cs shows the greater ionic radius, low first ionization energy, and electronegativity, while relatively average density and melting temperature among the A-site cations of niobates. Therefore, in the current study, we choose CsNbO_3_ (CNO) for additional pressure dependent analysis among different niobates. Very recently, pressure dependent vibrational, electronic, thermoelectronic, and optical properties of CNO have been reported^28^. However, the value of reported energy gap was quite low due to the use of conventional exchange correlational functional^28^. Despite the fact of very intriguing results, there is still a serious lack of knowledge regarding important aspects such as elastic properties, electronic band structure using advanced exchange correlation functional, charge density, and photocatalytic efficiency that must be addressed in order to take full advantages of CNO for potential technological applications.

In view of above circumstances, we explore structural, elastic, electronic, and photocatalytic properties of CNO. Investigating the effect of pressure on the aforementioned physical characteristics of CNO at low and high pressures is the main objective of this research.

## Methodology

Using the CASTEP algorithm, the first principles calculations of CNO were performed within the framework of density functional theory (DFT)^29^ using the plane wave pseudopotential approximation. Perdew-Burke-Ernzerhof (PBE)^30^ form of generalized gradient approximation (GGA) was used to calculate the exchange correlation energy generated by the electrical interaction of core ions and valance electrons in order to optimize the shape of the material. It is well known that the GGA-PBE approximation underestimates bandgap values, therefore, the electronic calculations were also performed using hybrid approaches such as sx-LDA^31^. After evaluating numerous cut-off points (where the energy is least), 400 eV was chosen as the plane-wave cut-off energy for the calculations. For Brillouin zone sampling, a Monkhrost-pack grid^32^ of 15 × 15 × 15 k-points was used. The calculation of the ground state atomic configuration were performed using the Broyden–Fletcher–Goldfarb–Shanno (BFGS) approach^33,34^ with the ultrasoft pseudopotential method^35^. The ultrasoft pseudopotential approach carries out the calculations of the materials by treating the electron density in the valance as the soft portion and the core region as the hard part, where the cut-off energy is significantly decreased and the computational efficiency noticeably improves. The stress–strain method included in the CASTEP code was used to determine the bulk modulus, shear modulus, and single crystal independent elastic constants. To execute the pseudo-atomic calculations, the following electronic configurations were taken into consideration: 5*s*^2^5*p*^6^6*s*^1^ for the Cs, 4*s*^2^4*p*^6^4*d*^4^5*s*_1_ for the Nb atom, and 2*s*^2^2*p*^6^ for the O atom. At the time of the geometry optimization, the energy convergence of the total energy, 0.5 × 10^−5^ eV/atom, the maximum interatomic force, 0.01 eV/Å, the maximum stress, 0.02 GPa, and the ionic displacement, 5 × 10^−4^ Å were set. CNO belongs to the cubic (space group ${\text{P}}\text{m-3m}$) crystal structure. The atomic positions for the Cs, Nb, and O atoms in the unit cell are (0, 0, 0), (0.5, 0.5, 0.5), and (0, 0.5, 0), respectively^36^.

## Results and discussions

### Structural properties

**Figure 1:**
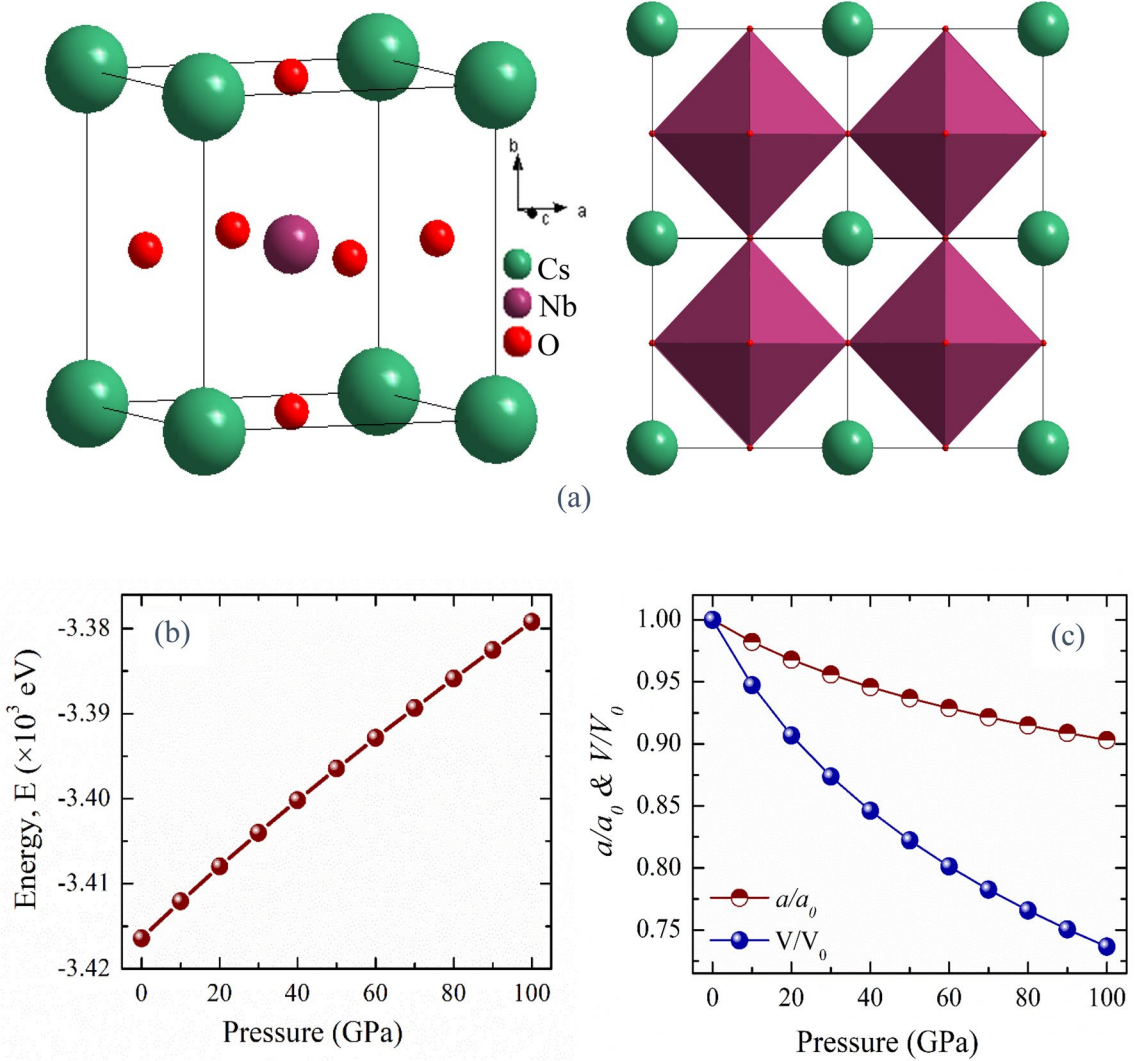
(**a**) 3D and 2D view of CsNbO_3_ (**b**) Pressure dependent ground state energy (E), and (**c**) Pressure dependent normalized lattice parameter,* a*/*a*_*0*_ and volume, V/V_0_ of CsNbO_3_.

Figure 1a shows the 3D and 2D schematic illustration of cubic CNO crystal structure. By fitting the reduced total energy of the crystal with respect to the volume of the unit cell using the Birch–Murnaghan equation of state^15^, the geometry optimizations were carried out to find the lattice constants, isothermal bulk moduli, and pressure derivatives. The obtained value of *a* = 4.15 Å, which is very close to the reported data of CNO^17^ that asserts the authenticity of the present calculation.

In order to investigate the pressure-dependent properties of CNO, the pressure dependent stability was checked. The calculated negative values of ground state energy of CNO reflects the stability of CNO up to 100 GPa. Figure 1b displays the pressure-dependent ground state energy of CNO. From Fig. 1b, it is seen that the minimum energy of CNO increase with increasing hydrostatic pressure. The rise in the ground state energy announces that the applied pressure drives the structure to be unstable and more energy is needed to stabilize the compound^37^.

Figure 1c demonstrates the fluctuation of the lattice constant and unit cell volume with hydrostatic pressures up to 100 GPa. From graph, it is realized that the normalized lattice constant and unit cell volume gradually decrease with increasing hydrostatic pressure. As the pressure rises, the structure's relative compression reduces as a result of the inter-atomic repulsive interaction.

### Stiffness constants and elastic moduli

**Figure 2:**
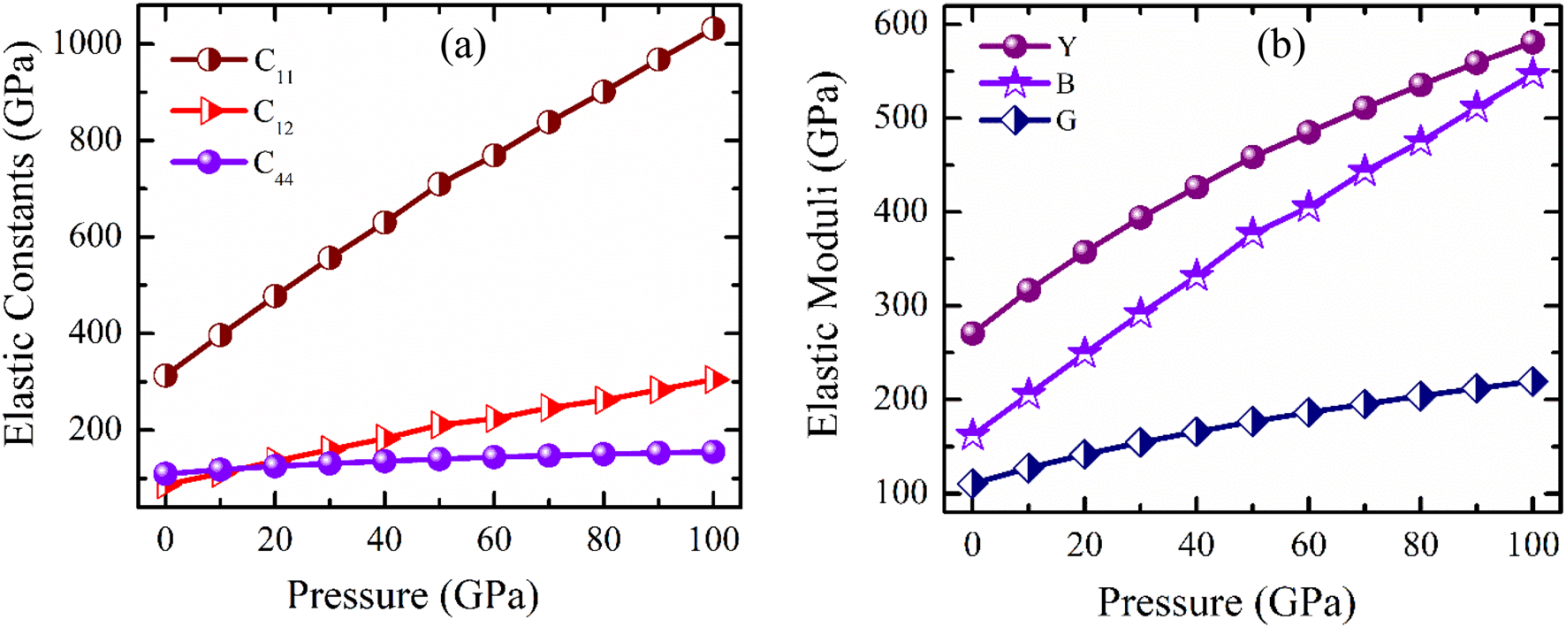
Pressure dependence of (**a**) Elastic stiffness constants and (**b**) Elastic Muduli of CsNbO_3_.

The "strain–stress" approach, which was used in earlier investigations^33,34^, was used to perform the calculations of elastic constants. The three independent elastic stiffness constants C_11_, C_12_, and C_44_ are obtained for CNO due to its cubic crystal structure. The pressure dependent fluctuations of C_ij_’s (i, j = 1, 2 & 4) which are shown in Table 2, and displayed in Fig. 2a.

The mechanical stability can be confirmed from the positively defined quadric form of energy density, where the coefficients obtained from DFT calculations (elastic stiffness) fulfill the cubic structure's fundamental minors and these are (C_11_–C_12_ > 0), (C_11_ + 2C_12_ > 0) and C_44_ > 0^32^. It is noticed that the elastic constants of CNO totally satisfy the aforementioned stability criteria for the entire range of applied hydrostatic pressure. Thus, CNO shows mechanical stability under pressure and the estimated elastic stiffness constant go under monotonous increase with increasing pressure.

The various elastic parameters, such as Y, B, G, and Poisson’s ratio (ν) and hardness (H_V_) were determined using the acquired elastic constants, C_ij_. By averaging the upper and lower bounds of Voigt's and Reuss's techniques, B and G have been calculated using the Voigt–Reuss–Hill approximation by the following equations:

1 $${\text{B}}_{\text{V}}={\text{B}}_{\text{R}}=\frac{1}{{3}}({\text{C}}_{11}+{{2}{\text{C}}}_{12})$$

2 $${\text{G}}_{\text{V}}=\frac{1}{{5}}({\text{C}}_{11}-{\text{C}}_{12}+{{3}{\text{C}}}_{44})$$

3 $${\text{G}}_{{\text{R}}}  = \frac{{5({\text{C}}_{{{\text{11}}}}  - {\text{C}}_{{{\text{12}}}} ){\text{C}}_{{{\text{44}}}} }}{{{\text{C}}_{{{\text{44}}}}  + 3({\text{C}}_{{{\text{11}}}}  - {\text{C}}_{{{\text{12}}}} )}}$$

4 $$\text{B} = \frac{{\text{B}}_{\text{V}}{+}{\text{B}}_{\text{R}}}{2}$$

5 $${\text{G}}=\frac{{\text{G}}_{\text{V}}{+}{\text{G}}_{\text{R}}}{2}$$

where, B_R_ and G_R_ indicate the Reuss approximation, B_V_ and G_V_ represent the Voigt approximation, and B and G have been calculated following the Voigt–Reuss–Hill approximation^38^.

Additionally, Y, ν and H_V_ are the function of B and G and can be calculated from the following equations

6 $$\text{Y} = \frac{\text{9BG}}{\text{3B+G}}$$

7 $$ \nu = \frac{{3{\text{B}} - 2{\text{G}}}}{{2\left( {3{\text{B}} + {\text{G}}} \right)}} $$

8 $$ {\text{H}}_{{\text{V}}} = {2}\left( {{\text{G}}^{{3}} /{\text{B}}^{{2}} } \right)^{{0.{585}}} - {3} $$

The calculated values of Y, B, and G under different pressure have been reported in Table 2. According to Table 1, the CNO has a large value of Y among the three elastic moduli (B, Y, and G) across the whole range of pressure. As the elastic tensor is directly proportional to Y, hence, the relatively large value of Y indicates that the CNO possesses a high elastic tensor. It can be also noticed that the elastic tensile resistance is getting increases with the increase of pressure and suggest the applicability of CNO at very high pressure.

**Table 1:** The lattice parameter (*a*) and unit cell volume (*V*) of CsNbO_3_.

Pressure (GPa)	*a* (Å)	*V* (Å^3^)	Reference
0	4.15	71.47	This work
	4.48	89.91	Ref.^17^
10	4.07	67.42	This work
	4.04	65.94	Ref.^28^
20	4.01	64.48	This work
	3.98	63.04	Ref.^28^
30	3.96	62.10	This work
	3.94	61.16	Ref.^28^
40	3.92	59.93	This work
	3.90	59.32	Ref.^28^
50	3.88	58.41	This work
	3.87	57.86	Ref.^28^

Again, Y is inversely proportional to the critical thermal shock coefficient inside the elastic limit, which has an impact on a solid's thermal shock resistance^39^. Better thermal shock resistance is represented by a lower value of Y. The value of Y for LiNbO_3_^40^, NaNbO_3_^41^ and KNbO_3_^42^ are 356, 272 GPa and 298 GPa respectively, whereas the obtained value for CNO is 271 GPa. As a result, CNO can be more suitable for thermal barrier coatings in comparison with above mentioned niobates. As demonstrated in Fig. 2b, it can be seen that G is less than B at all the pressure, and hence, rather than the volume change, shape deforming stress should be used to control the mechanical failure mechanism of CNO when pressure is applied.

### Ductility and hardness

**Figure 3:**
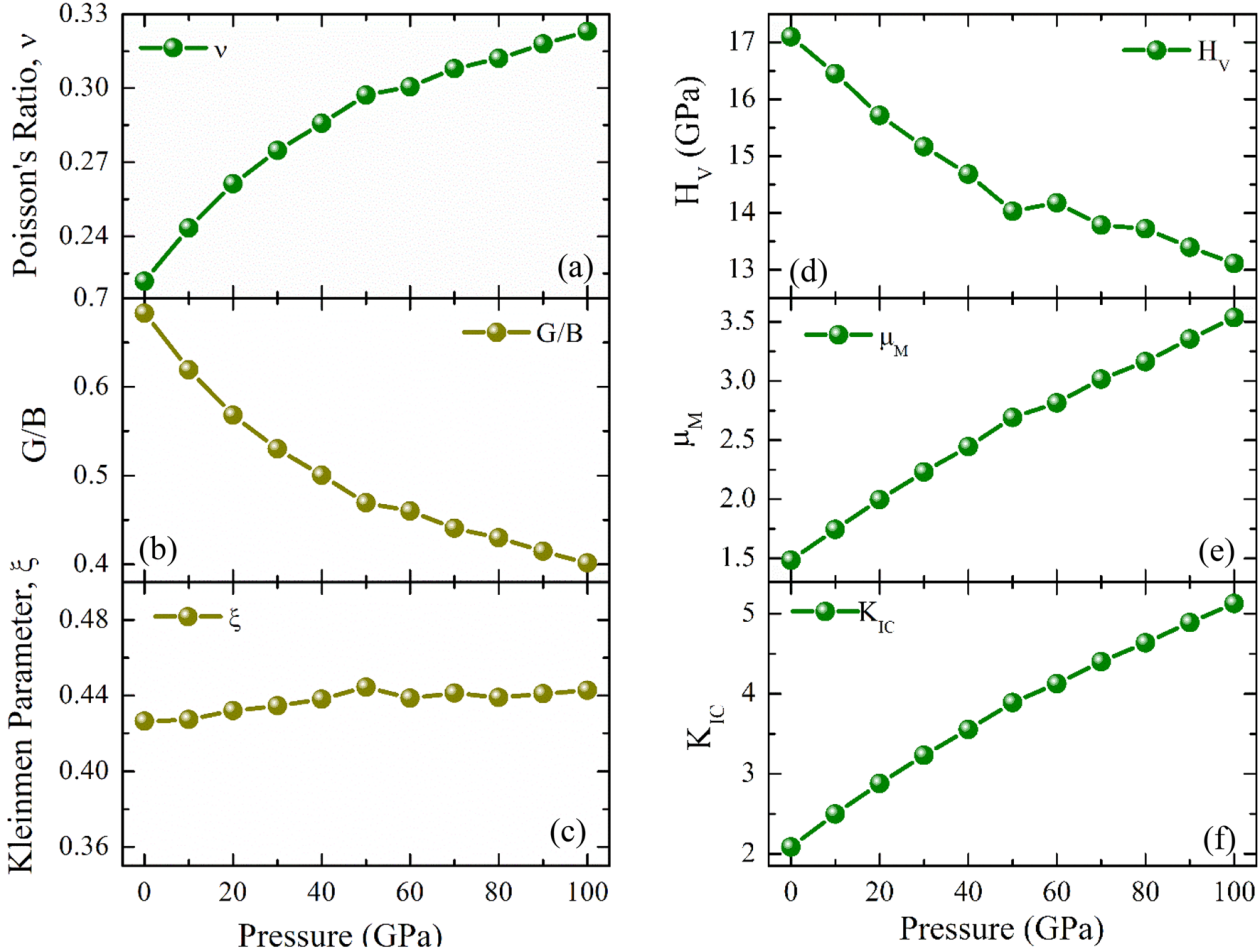
Changes of (**a**) Poison’s ratio, (**b**) Pugh’s ratio, (**c**) Kleinman parameter, (**d**) Vickers (**e**) machinability index, μ_M_, and (**f**) fracture toughness, K_IC_ hardness under pressure of CsNbO_3_.

The hardness of materials can be accurately predicted with significant support from Pugh's (G/B), Poisson's (ν) ratio and Cauchy Pressure (CP). Frantsevich et al.^43^ first reported the separation of ductility from brittleness of materials on the basis of Poisson’s ratio. Frantsevich rule suggested that ν ~ 0.26 as the border line which separates the brittle from ductile character. If the Poisson’s ratio is greater than 0.26 then the material will go under plastic deformation (ductile) otherwise the material will fracture having no plastic deformation (brittleness). On the other hand, Niu et al.^44^ have shown that one can draw a line on the basis of the Pettifor’s criterion^45^, that intersects the Pugh’s criterion at G/B = 0.571. The value of Pugh’s ratio less than 0.571 predict the ductile nature while the G/B is greater than 0.571 suggest the brittle nature. For cubic crystals, the difference between (C_12_–C_44_) is known as Cauchy pressure. If the value of C_44_ has a value greater than C_12_, according to Pettifor’s rule^45^, the materials with negative Cauchy pressure have covalence bonds and thus become brittle in nature. Otherwise, the materials have ionic bonding and thus become ductile. The values of ν, G/B and CP (Table 2) predicted that the material possesses ductile nature below 20 GPa, and its shows brittleness for ≥ 20 GPa which are shown in Fig. 3a and b. The Kleinman parameter (*ξ*) defied by $\xi = \frac{{{\text{C}}_{11} + 8{\text{C}}_{12} }}{{{\text{7C}}_{11} + 2{\text{C}}_{12} }}$ and essential parameter to determines the substantial contribution of the bond bending and bond stretching to minimize the external pressure effect whose value ranges from 0 to 1^46^. All the calculated values of ξ as shown in Fig. 3c forecast that CNO displays prominent bond bending over bond stretching at all external pressure.

**Table 2:** Elastic stiffness constants, *C*_*ij*_ in GPa, Elastic moduli (*B*, *G*, *Y*) in GPa, Pugh’s ratio (*G/B*), Poisson’s ratio (*ν*), Cauchy pressure (*C*_12_*–C*_44_) in GPa, Kleinman parameter (*ξ*) and Vickers hardness (*H*_*V*_) in GPa of CsNbO_3_ under pressure, P (GPa).

P	*C*_11_	*C*_12_	*C*_44_	*B*	*Y*	*G*	*G/B*	*ν*	*C*_12_*–C*_44_	*ξ*	*H*_*V*_
0	312	87	109	162	271	111	0.683	0.221	− 22	0.426	17.10
10	396	110	118	206	317	127	0.619	0.243	− 7	0.427	16.45
20	478	135	125	249	357	142	0.568	0.261	10	0.432	15.71
30	556	159	131	292	394	155	0.530	0.275	29	0.434	15.16
40	630	183	136	331	427	166	0.500	0.286	47	0.438	14.68
50	710	211	140	377	459	177	0.469	0.297	71	0.444	14.03
60	769	223	144	405	486	186	0.460	0.301	79	0.439	14.18
70	838	246	147	444	511	195	0.441	0.308	99	0.441	13.78
80	901	262	150	475	536	204	0.430	0.312	112	0.439	13.72
90	968	284	153	512	559	212	0.415	0.318	131	0.441	13.40
100	1032	305	154	547	581	220	0.401	0.323	150	0.443	13.11

The pressure dependent calculated values of Vickers Hardness of CNO have been listed in Table 2 and shown in Fig. 3d. A decreasing trend of hardness can be observed for the increasing pressure because the lower value of G/B and the larger value of Poisson’s ratios are the indication of less hardness of the material and vice-versa^47^. The high value of H_V_ predicts the CNO is a hard material and useful for mechanical applications.

The stress intensity factor K_IC_ can be used to calculate the quantitative value at which a tiny break in the material starts to spread. A reliable model of fracture toughness was proposed by Niu et al.^44^ and used the following empirical formula to calculate K_IC_:

9 $$ {\text{K}}_{{{\text{IC}}}} = {\text{ V}}_{0}^{{{1}/{6}}} {\text{G}}\left( {{\text{B}}/{\text{G}}} \right)^{{{1}/{2}}} $$

where V_0_ is the volume per atom.

From the calculation, it can be seen that CNO possesses a high value of K_IC_ in the whole range of pressure (Fig. 3f) i.e., the material can show high resistance to fracture propagation or micro cracks.

The machinability index, μ_M_ (= B/C_44_)^48^ is important for determining how lubricating and plastic a solid is^49^. Figure 3e shows that the value of μ_M_ increases in accordance with increasing pressure that proves the high-pressure applicability of CNO. In comparison to other materials, CNO^48^ shows a greater value of μ_M_, which indicates excellent lubricating qualities and a reduced friction value that may be useful to design industrial machineries.

### Electronic properties

**Figure 4:**
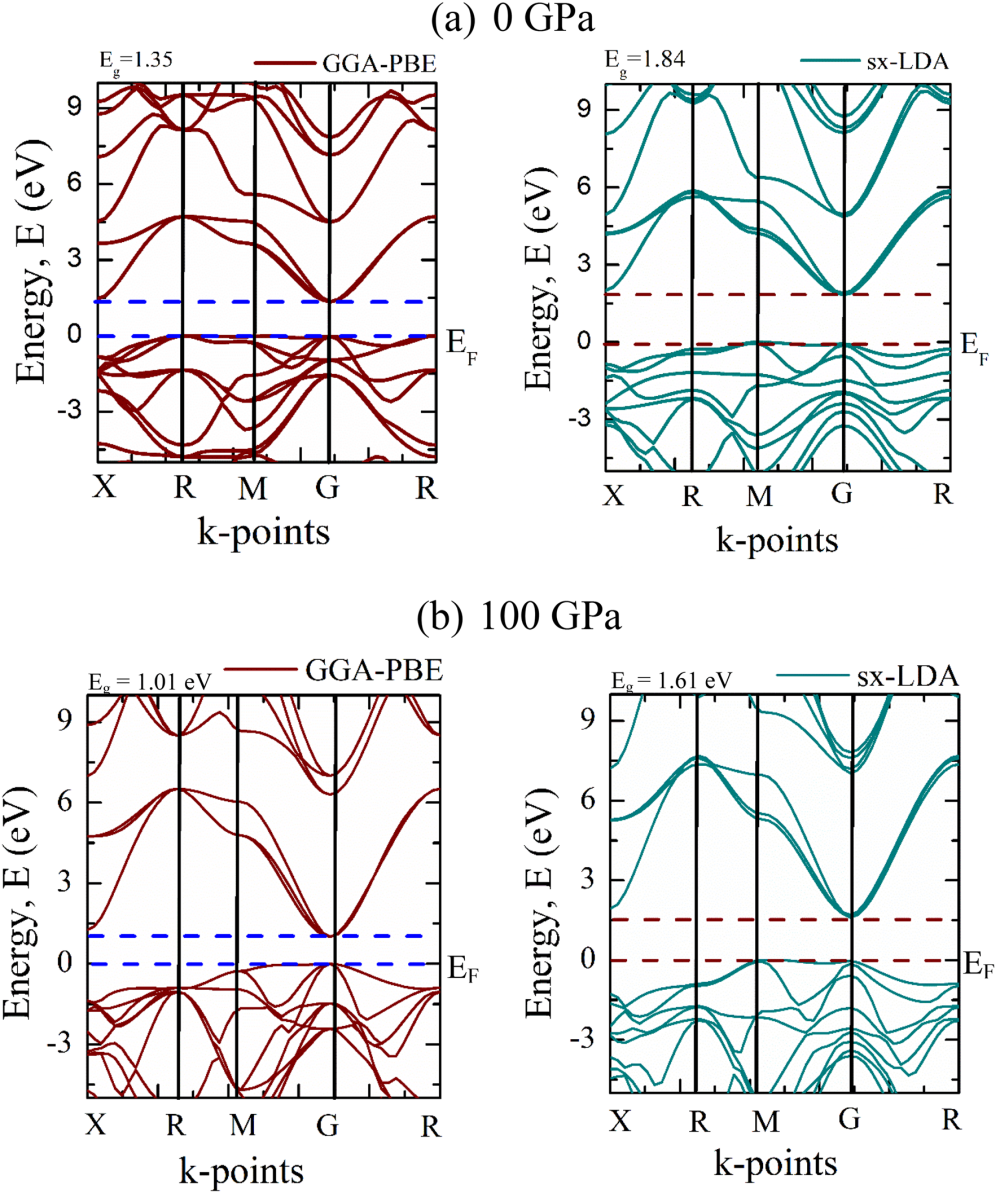
Electronic band structures of CsNbO_3_ calculated by using GGA-PBE, and sx-LDA approximations at (**a**) zero pressure and (**b**) 100 GPa.

We investigate the electronic band structure of CNO (Fig. 4) that describes the state of electrons in terms of their energy, E, and momentum, k. Figure 4 shows that CNO exhibits semiconducting nature with an indirect bandgap. In general, indirect bandgap semiconductors are utilized for photovoltaic device applications^17^.

This simulated bandgap is found to be fitting well with the prior theoretical value^13^. As it is well known that the GGA-PBE approach underestimates the bandgap values, therefore, we have performed the calculation using hybrid approaches such as sx-LDA since it gives the bandgap value close to the experimental results^31^. Interestingly, the bandgap value is enhanced significantly to 1.84 eV due to the use of sx-LDA (Fig. 4). Unfortunately, there is no available experimental data to compare the present result. The bandgap of CNO predicts the IR photodetector applications and substantiates the photoactive characteristics.

The electronic band structure also determines the dispersion relation for electrons in a material. The G–R direction shows the larger energy dispersion in comparison with X–R, R–M, and M–G directions for CNO. Consequently, it is believed that the band dispersion in the G–R direction controls the charge density^50^. Highly dispersive bands correspond to higher electrical conductivity due to their low carrier effective mass and higher charge mobility^51^.

**Figure 5:**
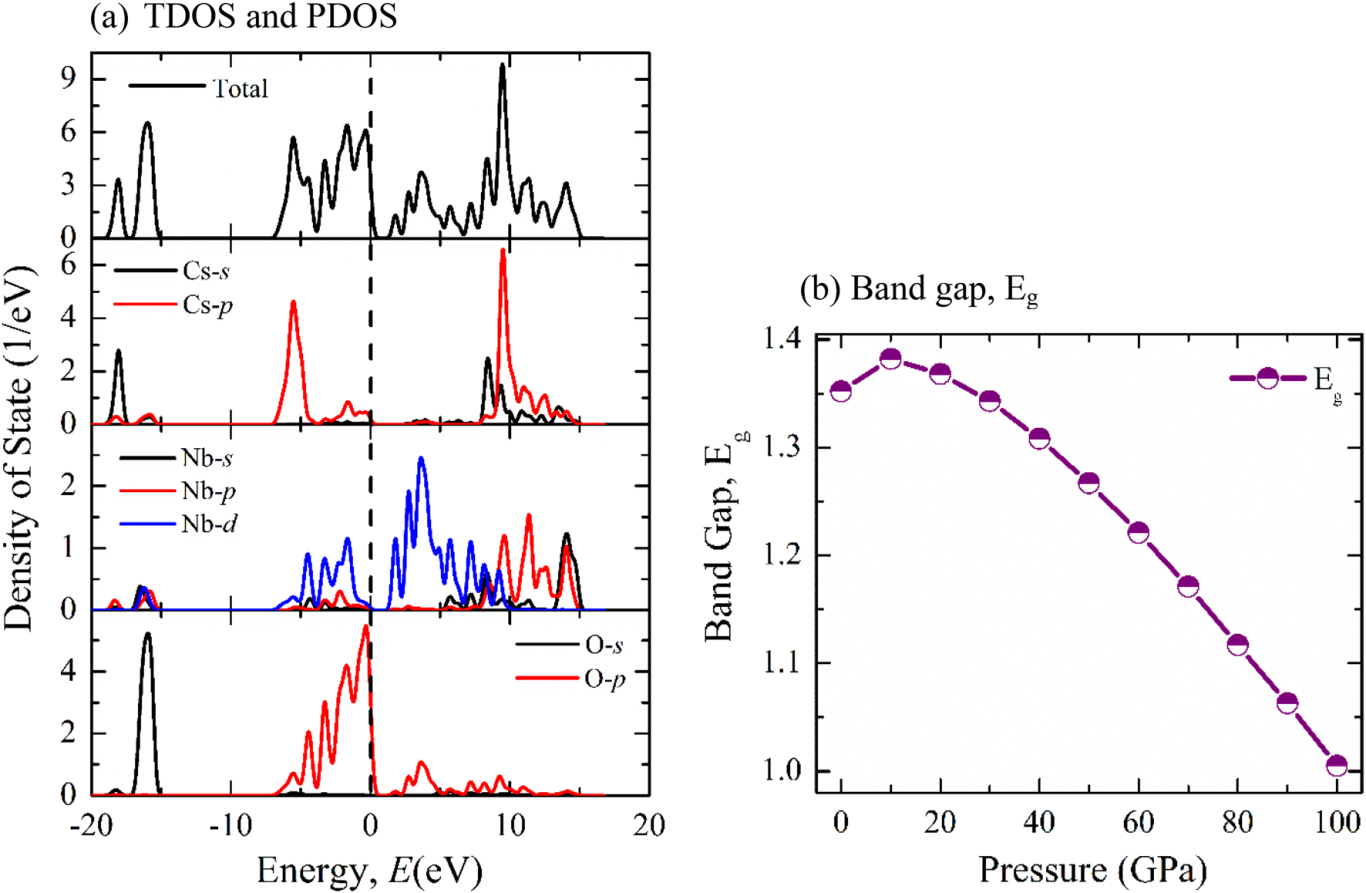
Graphical representation of (**a**) Total and partial DOS at zero pressure, and (**b**) Pressure dependent change of bandgap of CsNbO_3_.

Figure 5a depicts the total and partial density of states of CNO to analyze the orbital contribution close to the Fermi level. As displayed in Fig. 5a, below the Fermi level (0 to − 6.9 eV), the valance band (VB) arises mainly from the contribution of 2*p* orbital of O atom, which is well-known for oxide-based semiconductor^52^. Strong hybridization among Cs-5*p*, Nb-4*d*, and O-2*p* orbitals is also observed in the top of VB. Besides, O-2*p*, and Nb-4*d* orbitals are mainly attributed to the bottom of the conduction band (CB). Cs-5*p*, Nb-4*p* orbitals also contribute to the upper CB.

The bandgap, preferred orientation, and thermoelectric characteristics of CNO are all significantly changes with the external pressure. The obtained values of E_g_ using conventional GGA method at 0 and 100 GPa are 1.35 eV and 1.01 eV, respectively. On the other hand, when we use the hybrid functional like sx-LDA, the bandgap values at 0 and 100 GPa are 1.84 eV and 1.61 eV, respectively. The change of E_g_ for GGA and sx-LDA are 0.34 eV and 0.23 eV which is very closed. However, it is well known that the hybrid functional gives more accurate value (closed to experimental result) of bandgap compared to conventional functional. A material to be a narrow bandgap semiconductor, the value of the bandgap should be near the infrared region. The obtained value of the bandgap using hybrid functional suggests that CNO can not be a narrow bandgap semiconductor at 100 GPa.

It can be seen from Fig. 5b that the bandgap of CNO increases up to 10 GPa and after that it begins to decrease with increasing pressure. Because electron–hole pairs become closer under pressure and their Coulomb interaction cannot be overlooked initially, the bandgap widens, leading to increase kinetic energy. Further increased pressure with increasing kinetic energy, causing the change in electron/hole-ion potential, makes the material able to neglect the Coulomb interaction and impose the electronic state to be closer. As a result, the E_g_ decreases later with the increasing pressure. This observation is well agreed with recent theoretical report^28^, however, the authors do not describe the phenomena below 10 GPa.

Mulliken population analysis provides details on the charge, bond length, and bond population in a solid crystalline system, which aids in figuring out how charges are distributed among the bonds as well as ionicity and covalency of a material. The bonding and antibonding states, respectively, are responsible for the positive and negative bond overlap populations. The calculated data of charge, population, and bond length under pressure of CNO have been reported in Table 3.

**Table 3:** Calculated pressure dependent values of Mulliken effective charge (e), bond population and bond length (Å) of CsNbO_3_.

P (GPa)	Mulliken effective charge (e)			Bond population		Bond length (Å)	
	O	Nb	Cs	O–Nb	O–Cs	O–Nb	O–Cs
0	− 0.77	0.93	1.38	1.26	− 0.38	2.07	2.93
10	− 0.79	1.04	1.32	1.20	− 0.35	2.04	2.88
20	− 0.80	1.13	1.26	1.14	− 0.33	2.01	2.84
40	− 0.82	1.29	1.17	1.03	− 0.28	1.96	2.77
60	− 0.84	1.42	1.09	0.93	− 0.24	1.93	2.72
80	− 0.85	1.54	1.01	0.82	− 0.20	1.90	2.68
100	− 0.85	1.67	0.92	0.72	− 0.16	1.87	2.65

The reported data in Table 3 predicted that in CNO the Nb (0.93) shows a lower atomic charge contribution to form an atomic bond with the O atom. The Nb–O (1.26) bond also provides the higher value of bond populations than that of the Cs–O bond. The Nb–O (2.07 Å) bond length is also shorter than the Cs–O bond length. These observation refer to the strong covalent characteristics of the Nb–O bond. The antibonding situation of the Cs–O bond can be confirmed by the reported (− 0.38) value. The applied pressure bring change to the hybridization, charge, and bond population along with the bond length. From Table 3, it can be seen that with increasing pressure the covalency of Nb–O bond is decreased. In contrast, the antibonding character of the Cs–O bond is becoming weaker as the applied pressure give rise to the bond populations and reduces the charge of the Cs atom.

**Figure 6:**
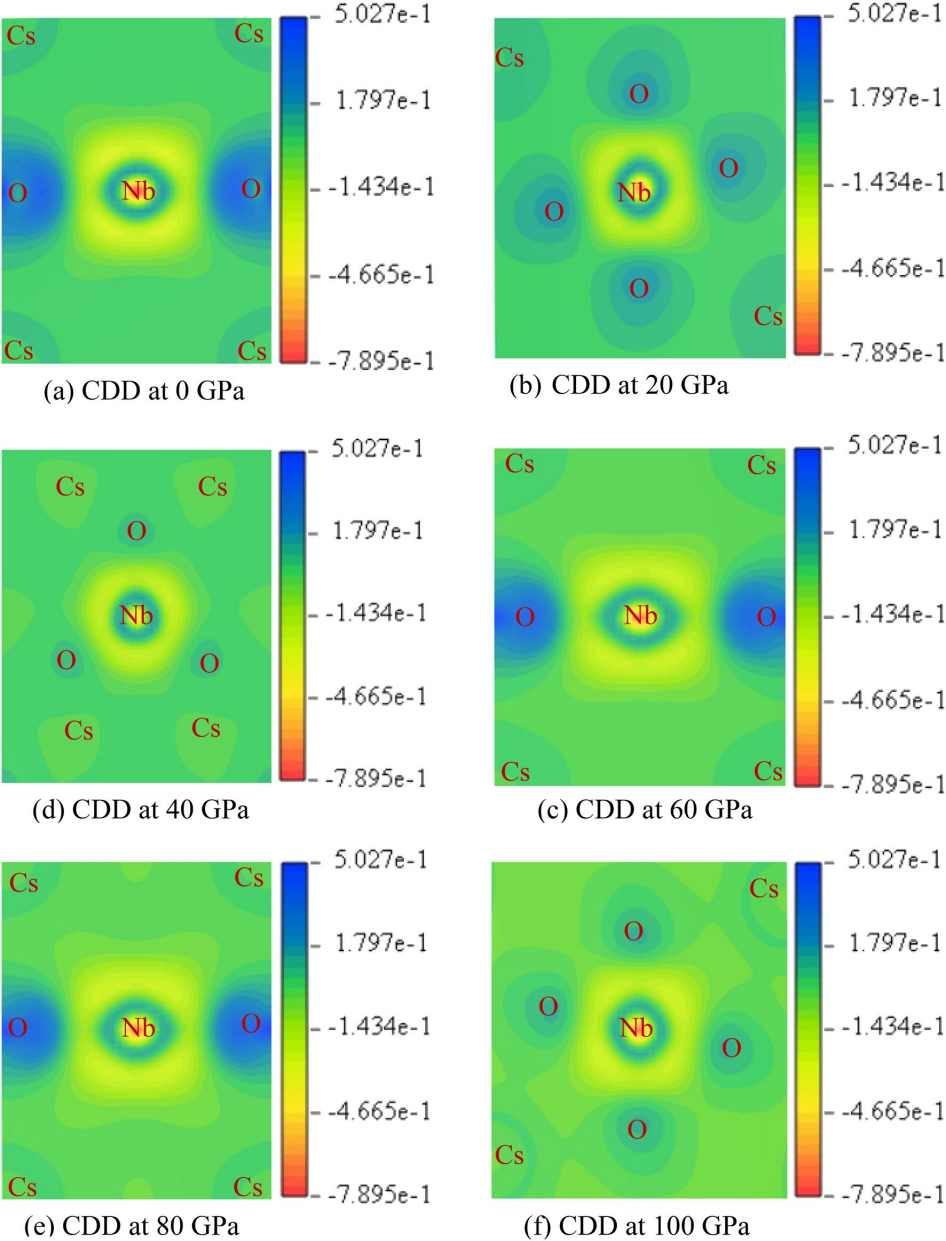
The electronic charge density (CDD) map of CsNbO_3_ at (**a**) zero pressure, (**b**) 20 GPa, (**c**) 40 GPa (**d**) 60 GPa (**e**) 80 GPa and (**f**) 100 GPa.

Understanding the type of atomic bonding in a compound can be done by looking at the contour map of the electron charge distribution among the atoms that make up the compound. Figure 6a–d show the contour color map of CNO at 0, 20, 40, 60, 80 and 100 GPa. The electron cloud that has been generated by the charge distributions around the atoms is almost spherical, and its intensity controls how much charge is accumulating. In Fig. 6a, it can be seen that the maximum electron density has been grown around the O-atom that exhibits strong electron localization. With increasing pressure the electron density increase around the O-atom that consistent with Mulliken Population analysis that gives rise to the antibonding character of the O–O bond. This increased electron density is also responsible for the lowering strength of the covalent Nb–O bond. As the positive charge density around the Cs atom decreases, the antibonding character of the Cs–O bond becomes weaker.

The antibonding nature can be explained from the correlation of DOS, band structure and CDD map. In the band structure, it is seen that the dispersion curve in the valence band is flat at a finite wave vector. It indicates the valance band to be occupied by the electrons with same wave vector and in CNO, electrons of Cs-5*p*, O-2*p *orbitals are responsible which are observed from partial density of states (Fig. 5). With the same wave vector, the wave functions of the electrons from different orbital interfere each other. From the Pauli’s exclusion principal, if two electrons try to share an identical set of quantum numbers, their probability functions cancel (destructive interference) and create zero electron probability density between the orbitals. As a result, from CDD, we observe the antibonding nature of Cs-5*p* and O-2*p* orbitals. On the other hand, due to the constructive interference of the wave functions of Nb-4*d* and O-2*p* suggest the bonding nature.

### Effective mass and photocatalytic activity

**Figure 7:**
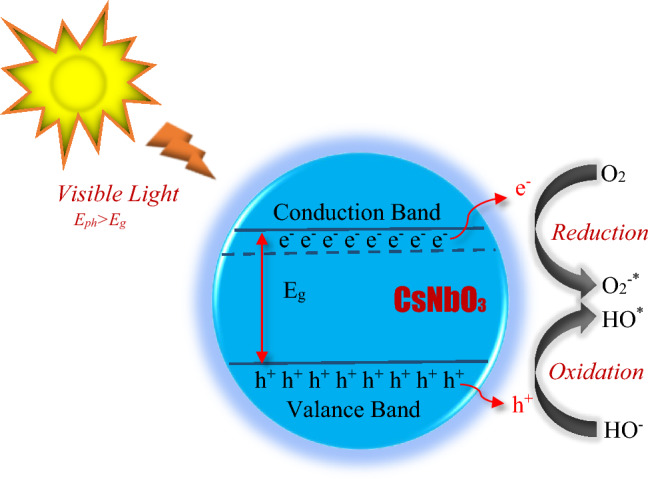
Schematic illustration of photoactivation and charge carriers transfer in CsNbO_3_.

To expand light absorption into the visible area and effectively use the solar energy, an efficient photocatalyst needs a bandgap energy less than 3 eV^53^. The bandgap of the semiconducting CNO proves that it can absorb a wide range of solar radiation. In Fig. 7, E_ph_ represents the energy of the photon and the E_ph_ needs to be greater than E_g_. The indirect bandgap (Fig. 4) may be more advantageous for photocatalytic applications because it may lower the radiative recombination rate due to the momentum mismatch between the CBM and VBM, which is advantageous for a longer carrier lifetime and therefore, photocatalytic activity^54^. The band structures in Fig. 4 also make it clear that the CBM of CNO is more parabolic (highly dispersive) than the VBM. This suggests that the photogenerated electron may have a lower effective mass than the hole effective mass.

The rate of diffusion and separation efficiency of the photogenerated carriers have a significant impact on a material's photocatalytic activity^55^. A high ratio of electron (${\text{m}}_{\text{e}}^{*}$) to hole (${\text{m}}_{\text{h}}^{*}$) effective mass, results a low recombination rate and a large difference between electron–hole mobility. “The E–k relation at the conduction band minimum can be well fitted by a parabolic relation of the form:

10 $${\text{m}}_{\text{e}}^{*}={\hbar }^{2}\left({\frac{{{\text{d}}^{2}{\text{E}}}}{{{\text{dk}}^{2}}}}\right)^{-1}$$

where, E is the band-edge energy and k is the amplitude of reciprocal lattice vector. After calculating the second order derivative from the minimum of the conduction band of E–k diagram, the effective mass of electron is obtained.” From the band diagram of GGA-PBE approximation, the ${\text{m}}_{\text{e}}^{*}$ is estimated at G-point to be 0.67 m_e_ in CBM, whereas the ${\text{m}}_{\text{h}}^{*}$ at R-point is estimated to be 2.63 m_e_ in VBM. Usually ${\text{m}}_{\text{h}}^{*}$ is positive, while ${\text{m}}_{\text{e}}^{*}$ is negative. For sx-LDA approach, the ${\text{m}}_{\text{e}}^{*}$ has been calculated at G-point in the CBM is 0.61 m_e_ whereas the estimated values of ${\text{m}}_{\text{h}}^{*}$ at M-point is 4.40 m_e_ in CBM. It is noted that electron and hole effective masses of CNO is close to ZnO^56^, BaTiO_3_^2^ and RbSr_2_Nb_3_O_10_^19^ and lower than TiO_2_^57^ and In_2_O_3_^58^ semiconductor photocatalysts. In present work, the low values of ${\text{m}}_{\text{e}}^{*}$ and ${\text{m}}_{\text{h}}^{*}$ imply that it has high electrical conductivity. The photogenerated carriers can quickly transfer to the surface of CNO, which is crucial for photocatalytic activity. Nevertheless, the low value of hole carrier effective mass (2.63m_e_ for GGA-PBE) has a great impact on catalytic efficiency. The low value of carrier effective mass of the hole causes the highest rate of H_2_ production. In addition, relatively high ratio of electron (${\text{m}}_{\text{e}}^{*}$) to hole (${\text{m}}_{\text{h}}^{*}$) effective mass is associated with easy separation of photogenerated electrons from their associated holes, which lowers the probability of their recombination and increases their availability to perform catalytic processes. This is because a decrease in carrier mass results in a corresponding increase in carrier mobility. Therefore, the reduction of recombination of the electron–hole carrier of CNO enhances its photocatalytic efficiency.

In order to clarify the separation of photogenerated electron–hole pairs over the CNO, it is necessary to find out the conduction and valence band edge potentials of the component. In the case of water splitting, the valence band edge potential should be more positive than the potential needs for oxygen evolution (1.23 eV vs. *NHE*) and the conduction band potential should be more negative than the potential corresponding to the hydrogen evolution reaction (0 eV vs. *NHE*). These energy band potentials are calculated using the following empirical equations^59^:

11 $$ {\text{E}}_{{{\text{CB}}}} = \chi - {\text{E}}_{{\text{e}}} - \frac{1}{2}{\text{E}}_{{\text{g}}} $$

12 $${\text{E}}_{\text{VB}}={\text{E}}_{\text{CB}}{+}{\text{E}}_{\text{g}}  $$

where, χ is the absolute electronegativity of CNO and the E_CB_ and E_VB_ indicate the band edge potentials of CB and VB, respectively. The Mulliken electronegativity of an atom is determined by the average value of its electron affinity and its first ionization energy^60^. E_g_ denotes the acquired electronic bandgap, while E_e_ resembles the energy of free electrons on the hydrogen scale (4.5 eV).

**Figure 8:**
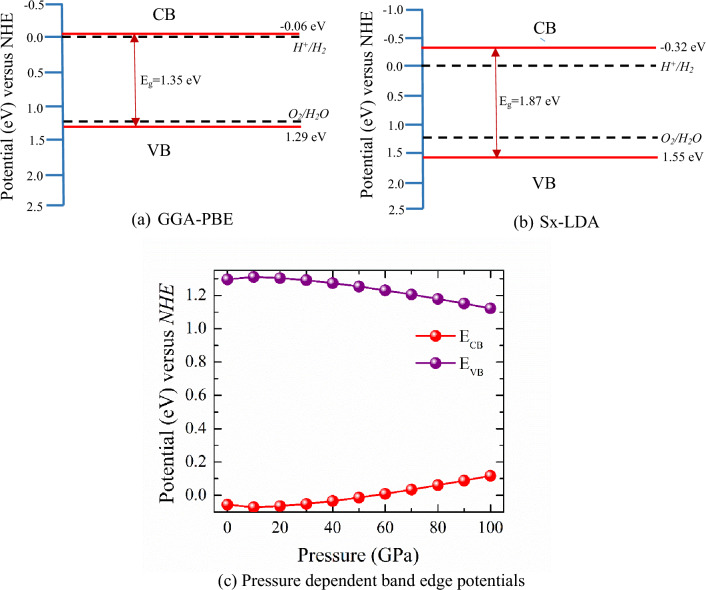
Calculated potentials for conduction band minimum, E_CB_, and valence band maximum, E_VB_ using the band structures and bandgap values calculated by (**a**) GGA-PBE, and (**b**) sx-LDA, and (**c**) Pressure dependent changes of E_CB_ and E_VB_ for CsNbO_3_.

The CNO has an estimated electronegativity, χ of 5.12 eV. Figure 8a and b show the band edge potentials of VB and CB in CNO for the GGA-PBE, and sx-LDA approximations. The thermodynamic aspect of the CBM potential of CNO, which is negative, demonstrates that H^+^ to H_2_ reduction will occur which could delay the electron–hole recombination process. As the dilation in the recombination process enhances the photocatalytic efficiency, the negative value of CB potential substantiates the CNO to be a good photocatalytic material. The evolution of O_2_ from water is revealed by the VBM potential, which is higher than O_2_/H_2_O (1.23 eV) in all approaches. The larger value of the VBM potential of CNO indicates the possibility of a spontaneous photocatalytic reaction. Therefore, the VBM potential also proves the CNO is good for water splitting applications.

The band edge potentials that characterize the photocatalytic activity are greatly influenced by the bandgap as shown in Fig. 8c. With increasing pressure, the CB potential initially decreases (becomes more negative) and then increase (after 10 GPa) and exits the potential of hydrogen evaluation reaction i.e., the CNO losses its photocatalytic efficiency. The change of VB potential under pressure also displays a similar effect on photocatalytic activity. The change in the bandgap, E_g_ is responsible for these changes. However, from these calculations, it can be confirmed that at 10 GPa CNO shows the highest photocatalytic efficiency and at high pressure, it stops functioning as a photocatalyst.

## Conclusions

Using the state of art density functional theory, we investigate the effect of hydrostatic pressure on the structural, elastic, electronic, and photocatalytic properties of CsNbO_3_. The values of Pugh’s ratio, Poisson’s ratio, Cauchy pressure, and Kleinman parameter confirmed the ductile nature below 20 GPa. The obtained high value of fracture toughness and machinability index revealed the good mechanical application against micro-cracks and excellent lubricating qualities. The electronic band structure discloses the semiconducting character of the compound. Two different approaches provide variety in bandgap energy of 1.35 and 1.87 eV for GGA-PBE, and sx-LDA, respectively. The estimated indirect bandgap suggests the good photoactive character of CsNbO_3_. Under pressure, the bandgap energy increases initially till 10 GPa and then decreases gradually with pressure. The calculated Mulliken effective charge, bond population and bond length revel the good covalency and antibonding character of Nb–O and Cs–Nb, respectively. The calculation of electron charge density was in good agreement with the Mulliken population analysis. Low value of carrier effective mass assert that CsNbO_3_ has to be good electrical conductivity as well as photocatalytic activity. The calculated band edge potentials revealed the CNO would exhibit excellent water splitting efficiency. The result of the current computations are expected to serve as a foundation for future experimental and theoretical investigations of the suitability of CsNbO_3_ in various device applications.

## Data Availability

The authors ensure that the data supporting the findings of the study are available to M. Monira (muniramarjanum@gmail.com) and can be provided if needed.
